# Psoas muscle quantified muscle status and long-term mortality after cardiovascular interventions

**DOI:** 10.1080/07853890.2023.2259798

**Published:** 2023-09-22

**Authors:** Otto Järvinen, Juho T. Tynkkynen, Marko Virtanen, Pasi Maaranen, Iisa Lindström, Damir Vakhitov, Jari Laurikka, Niku K. Oksala, Jussi A. Hernesniemi

**Affiliations:** a Faculty of Medicine and Health Technology, Tampere University, Tampere, Finland; b Finnish Cardiovascular Research Centre Tampere, Tampere, Finland; c Centre for Vascular Surgery and Interventional Radiology, Tampere University Hospital, Tampere, Finland; d Heart Hospital, Tampere University Hospital, Tampere, Finland

**Keywords:** Sarcopenia, muscle mass, psoas muscle area, psoas muscle density, mortality

## Abstract

**Background** Psoas muscle area (PMA) and density (PMD) measured using computed tomography are potential surrogates for evaluating sarcopenia-related mortality risk among patients undergoing cardiovascular interventions. However, the optimal measurement method remains unclear.

**Methods** A retrospective registry study of three observational cohorts comprising 2248 patients undergoing cardiovascular interventions (*n* = 828 for abdominal aortic aneurysms, *n* = 983 transcatheter aortic valve implantations, and 437 patients undergoing open surgery for thoracic aortic and aortic valve pathology) was conducted. Age-independent associations between mortality and PMA, PMD, PMA indexed to height or body surface area, and the combination of PMA and PMD (lean PMA) were meta-analyzed (using individual participant data) across cohorts after sex stratification with median follow-up times ranging from three to five years for each cohort.

**Results:**

In the meta-analysis, psoas muscle measurements were significantly associated with mortality among men (*p* < 0.05), with high heterogeneity in the associations across all cohorts. There was very little difference in the association between PMA and PMD and mortality (HR 0.83, 95% CI 0.69–0.99, *p* = 0.002; HR 0.85, 95% CI 0.77–0.94, *p* = 0.041 for one SD increase in PMA and PMD in the random effects model). Combining PMA and PMD into one composite variable by multiplying their values together showed the most robust association in terms of the magnitude of the effect size in men (HR, 0.77; 95% CI 0.73–0.87, *p* < 0.001). Indexing PMA to body size did not result in any significant differences in this association. Among women, psoas muscle measurements were not associated with long-term mortality in this meta-analysis.

**Conclusions:**

Different psoas muscle measurements were significantly and very similarly associated with mortality among men but not among women. No single measurement stands out, although combining PMA and PMD seems to be a slightly stronger estimate in terms of effect size and should be considered in further studies.

## KEY MESSAGES

Significant sarcopenia affecting survival in patients undergoing heavy invasive operations may be preoperatively assessed using images of psoas muscle (PM) from routine computerized tomography but the optimal method for evaluation is unclear.

A meta-analysis of individual participant data of over two thousand patients undergoing cardiovascular interventions shows that different PM measurements of surface area and density were significantly and very similarly associated with mortality among men but not among women.

Combining PM area with PM density to one estimate of lean psoas muscle area seems to provide the strongest hazard estimate among men.

## Introduction

Sarcopenia, which is related to frailty, is defined as skeletal muscle mass loss, and a weakening disorder is associated with an increased likelihood of physical disability and mortality in the elderly [1–3]. In recent years, sarcopenia has also been found to independently predict postoperative survival among patients who undergo complex vascular, cardiac, or oncological surgeries and treatments [4–8]. The general condition of hospitalized patients deteriorates with increasing mean age in many populations globally, and it is important to provide clinicians with a clear and unanimously reproducible method to measure sarcopenia.

In recent years, several methods for determining the level of sarcopenia have been demonstrated. Muscle function can be assessed by testing handgrip strength, walking, standing balance, and chair raising time. The muscle mass can also be estimated using bioelectrical impedance analysis (BIA), anthropometric measurements, or radiological imaging. These parameters are often adjusted for body mass index, height, or body surface area [3, 9, 10]. The psoas muscle measurements assessed from an abdominal computed tomography (CT) scan have been found to correlate well with the level of sarcopenia and to act as a prognostic factor for postoperative survival [4, 11–14]. The replicability of measurements of psoas muscle (surface) area (PMA) and density (PMD) from routine preoperative images is shown to be excellent, making it an attractive subject for research [15].

The foremost difficulty in evaluating sarcopenia-related mortality risk based on the results of previous studies is the wide variation (or heterogeneity) in the definition of sarcopenia and its measurement in each study. Several recent studies have used CT-based PMA, PMA indexed to body size (to height or body surface area [BSA]) or PMD measured using Hounsfield units [4–6, 8, 11–14, 16–27]. PMD is claimed to be a more accurate estimation of sarcopenia since it indicates fat-free muscle tissue quality [28–31] although it fails to account for muscle size. In addition, so-called ‘lean’ value - a product of PMA and PMD - has been presented to capture both the area and quality of the psoas muscle in a single measurement [15, 32]. In addition to different measurement methods, the lumbar spine level from which the measurement is made varies between previous publications, with the L3 and L4 levels being the most common sites for measurement [5, 13, 15, 20, 22, 27, 28, 30, 32].

This study aimed to evaluate which method of measurement (and from what lumbar vertebral level) predicts the best overall postoperative mortality among patients undergoing invasive cardiovascular interventions. The evaluation was performed by a meta-analysis of three consecutive series of patients treated for abdominal aortic aneurysm (AAA), patients treated for aortic stenosis by transcatheter aortic valve implantation (TAVI), and patients treated for thoracic aortic (TA) pathology.

## Methods

### Study design

This was a retrospective registry study combining data from three real-life patient cohorts of consecutive patients who underwent different vascular and valvular operations at Tampere University Hospital and Tays Heart Hospital. The study center is the only service provider for these operations in the limited geographical area of Pirkanmaa with a catchment area of 0.5million inhabitants. It consisted of three cohorts with a total of 2685 patients: 1053 patients undergoing AAA repair between 1999–2019 (643 using an endovascular graft and 410 with open surgery), 1100 patients undergoing elective TAVI for aortic stenosis between 2008–2020 and 532 patients undergoing open surgery for TA pathology between 2007–2020. These patient populations were selected for this study as all these patients had significant cardiovascular burdens with varying operative risk, and routine preoperative computed tomography imaging was available for the measurement of psoas muscle surface area and density. This study was approved by the Institutional Review Board of Pirkanmaa Hospital District (overseeing research in Tampere University Hospital). According to the Finnish legislature, further ethical board review was not required due to the retrospective nature of this study. This study adhered to the ethical guidelines of the Declaration of Helsinki.

**Figure 1.**
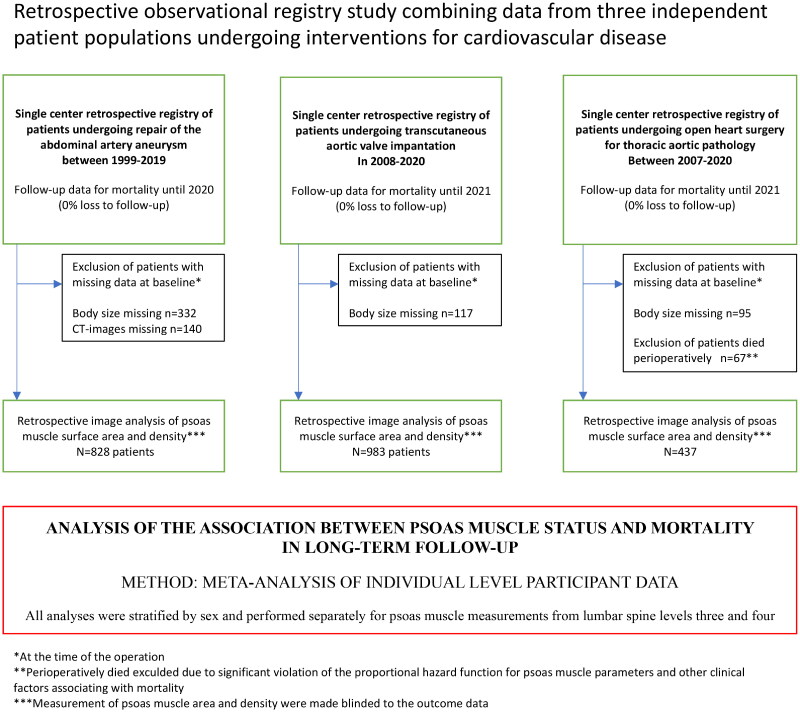
The overall study design.

Patients with missing psoas measurements from CT (140 patients treated for AAA) and missing body height and weight measurements (332 AAA patients, 117 TAVI patients, and 95 TA patients) were excluded. Finally, for patients who underwent surgery for ascending aortic pathology, there was a clear deviation from the proportional hazard assumption for various exposure variables regarding mortality, and all patients who died during the perioperative period (<28 days) were excluded (*n* = 67) from the analysis. In this cohort, the only significant predictors for perioperative mortality were urgency, by which the operation was performed, and kidney function before surgery. After exclusion, the final sample size for all three cohorts was 828 (AAA), 983 (TAVI), and 437 (TA), with a total of 2248. The overall study design is presented in Figure 1.

### Collection of clinical data

Clinical background data were acquired from two prospectively updated registries designed to collect relevant procedural and patient-related clinical data from patients undergoing invasive operations (the vascular surgery registry maintained by vascular surgeons and the KARDIO registry maintained by cardiologists and cardiothoracic surgeons). These data were further retrospectively updated using clinical information collected from the electronic hospital registry and by a full disclosure review of written patient records and charts.

### Psoas measurements

**Figure 2.**
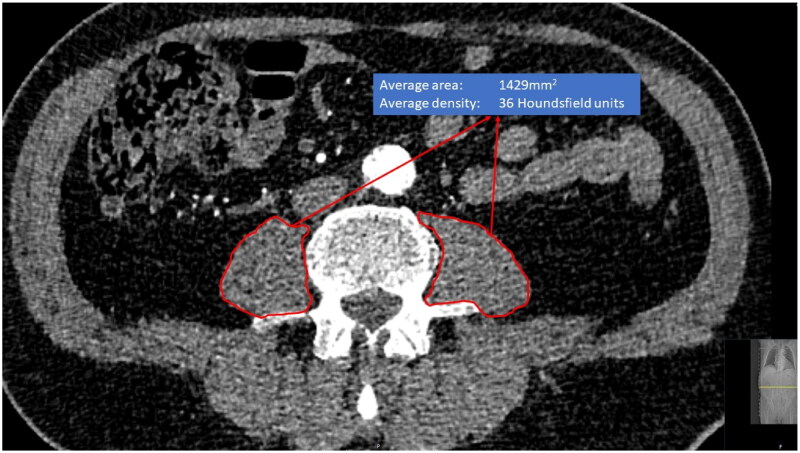
Measurement of psoas muscle area and density at fourth lumbar vertbrae level.

Psoas measurements were performed based on preoperative CT imaging and in some cases after the operation if only postoperative images were available (<3% of images taken within 30 days after the operation). Median and interquartile ranges for the time between CT imaging and the operation for different cohorts: 42 (13–49) days for AAA patients, 40 (17–74) for TAVI patients, and 49 (1–118) for TA patients. CT image measurements were performed in the contrast-enhanced arterial phase and axial slice thicknesses between 0.50–3.00 mm were used. Two different multidetector scanners were used: The General Electric LightSpeed 16-row scanner (GE Healthcare, Milwaukee, WI, USA) and the Philips Brilliance 64-row scanner (Philips, Cleveland, OH, USA) (∼80% with 100 kV and ∼20% with 120 kV). Both were in equal use and there was no selection between these scanners. CTA images were reviewed using medical imaging workstations (Carestream Vue PACS viewer version 11.4.0.1253, Rochester, NY, USA). The psoas muscles were carefully outlined with the free-hand tool by the authors of this study along the prominent muscle fascia after which the imaging workstation program automatically calculated the area and mean density of the outlined muscle (Phillips Intellivue software). Measurements of PMA in mm^2^ and PMD in Hounsfield units were collected. Measurements were made separately from the level of the L3 and L4 vertebrae below the upper endplate and approximately at the middle of the vertebrae, where both transverse processes were most visible (Figure 2). Left and right PMA and PMD measurements from both L3 and L4 levels were combined, and the average PMA and average PMD were calculated to produce an estimate of the average of both sizes, which is less prone to measurement error and reduces the number of required statistical tests. Preliminary quality control analysis did not show any significant differences in recorded psoas muscle parameters with different slice thickness. The reliability of the repeated measurement between clinicians is most frequently estimated as the intraclass correlation coefficient (ICC) [33]. The reproducibility of the outlined psoas muscle measurements measured by the intra- and inter-observer variability, is excellent [15].

### Outcome data

The *a priori* defined outcome in the analyses was the overall mortality (due to any cause) during the maximal follow-up period available in each cohort. Mortality data were available from the national mortality registry maintained by Statistics Finland, with no loss to follow-up.

### Statistical methods

Since psoas measurements were not complete for all study subjects, we performed all analyses with a single imputed dataset (R package MICE), and the number of patients with missing PMA and PMD measurements from either the L3 or L4 level, which had to be replaced with imputed values, are presented in Supplementary Table 1. For data imputation, all three cohorts were merged to improve data quality, and after imputation, cohorts were analyzed separately. Data imputation was performed based on sex, age, height, body surface area (BSA), cohort information, and available psoas measurements. The predictive mean matching method was used to impute the missing psoas measurements [34]. PMA and PMD measurements from the L3 and L4 levels were tested for mortality risk. PMA measurements, but not PMD measurements, were also indexed to body surface area (PMA/BSA) and height (PMA/height) and tested for mortality risk. All L3 and L4 measurements were sex- and cohort-specific before testing. In addition, L3 and L4 level-specific PMA, PMD, PMA/BSA, and PMA/height sum and product combinations with a maximum of two components were generated and tested against mortality risk. Before associations between product combinations were used, a detected minimum value of +1 was added to all scaled measurements to ensure that no negative values were used in multiplication. After the sum and product combinations were calculated, a new normal scaling was performed for all values. Hazard ratios (HR) were reported per 1SD change in the psoas value.

Associations between psoas measurements and mortality risk were analyzed separately in men and women using a Cox regression model adjusted for age. Adjustment for renal function did not significantly change the results, suggesting that the association between factors is independent of renal function, and only the age-adjusted results are presented. Subsequently, the predicted risk and area under the curve (AUC) were calculated for the different psoas measurements.

As we used three different study cohorts (AAA, TAVI, TA), all analyses were performed in a cohort specifically, and cohort-specific results were meta-analyzed using the inverse variance weight method. The heterogeneity in the meta-analysis was quantified using I^2^. The (PH) assumption was tested using the Schoenfeld residual test. The null hypothesis was rejected for values of *p* < 0.05.

## Results

### General characteristics at baseline and mortality in follow-up

Demographic data, risk factors, comorbidities, type and urgency of the operation, follow-up times, and mortality are presented in Table 1. The baseline age distribution, follow-up time, and overall mortality in each cohort varied substantially (Table 1). Patients treated for ascending aortic pathology had the lowest overall mortality (13.3% during a follow-up of 4.9 years [3.0–7.0]) and were youngest at baseline (65.0 years [53.0–71.0]) whereas the highest overall mortality was observed among patients undergoing AAA repair (55.80% mortality during a follow-up of 5.1 years [2.5–8.3]). The average PMA and PMD values for each cohort stratified by sex are presented in Table 2. The largest PMA and PMD values were observed among men in the TA cohort (the cohort with also the youngest mean age).

**Table 1.** Baseline characteristics of the patients in three cohorts.

	AAA *n* = 828	TAVI *n* = 983	TA *n* = 437
Follow-up years, median (IQR)	5.1 (2.5-8.3)	3.1 (1.9–4.7)	4.9 (3.0–7.0)
Dead at the end of follow-up, % (n)	55.8 (462)	36.6 (360)	13.3, 58
Emergency procedure	6.4%	9.9%^a^	22.7%^a^
Specific procedural details	Endograft in 70.3%^a^ Previous repair in 6.4%^a^	Transfemoral access in 88.8%	Aortic arch or descending aorta surgery in 19.8%^a^ Concomitant CABG in 15.2%^a^
Age (median, IQR)	74.5 (68.4–80.0)	82.0 (77.0–85.0)	65.0 (53.0–71.0)
Women, % (*n*)	11.2 (93)	52.6% (517)	26.5% (116)
Hypertension	65.7%	77.9%^a^	60.8%^a^
Diabetes mellitus	13.9%	32.0%^a^	9.8%^a^
Pulmonary disease	23.6%^a^	14.9%^a^	no data available
Dyslipidemia	48.1%	71.6%^a^	39.2%^a^
Coronary artery disease	46.9%^a^	13.7%^a^	15.2%^a^
Estimated glomerular filtration rate (mean, SD)	70.8 (21.1)	57.5 (19.8)	81.5 (20.3)
Body Mass Index (mean, SD)	26.95 (4.78)	28.08 (10.71)	27.93 (7.49)^a^
Body Surface Area (mean, SD)	1.96 (0.20)	1.85 (0.21)	1.99 (0.22)

AAA: abdominal aortic aneurysm repair; TAVI: transcutaneous aortic valve implantation; TA: thoracic aortic surgery; IQR: interquartile range.

^a^Data missing less than 10% of this information.

**Table 2.** The average PMA and PMD values for each cohort stratified by sex and p-values (Sig.) for significance for comparisons between cohorts in men and women (no statistical testing between men and women).

TAVI Mean (SD)		AAA Mean (SD)		TA Mean (SD)	
Men	Women	Men	Women	Men	Women
**Lumbar spine L3 level measurements for average psoas muscle area in mm^2^**					
860 (208)	599 (154)	770 (144)	568 (149)	1046 (215)	609 (180)
Sig.	Sig.	Sig.	Sig.	Sig.	Sig.
AAA <0.001	AAA0.106	TAVI <0.001	TAVI 0.106	TAVI <0.001	TAVI0.772
TA: <0.001	TA 0.772	TA <0.001	TA 0.078	AAA <0.001	AAA0.078
**Lumbar spine L3 level measurements for average psoas muscle density in Houndsfield units**					
34.9 (10.6)	37.7 (10.9)	33.6 (12.7)	32.4 (13.7)	42.0 (10.0)	39.0 (11.5)
Sig.	Sig.	Sig.	Sig.	Sig.	Sig.
AAA 0.126	AAA <0.001	TAVI 0.126	TAVI <0.001	TAVI <0.001	TAVI 0.405
TA <0.001	TA 0.405	TA <0.001	TA <0.001	AAA <0.001	AAA <0.001
**Lumbar spine L4 level measurements for average psoas muscle area in mm^2^**					
1152 (239)	819 (183)	1058 (196)	794 (169)	1404 (266)	981 (138)
Sig.	Sig.	Sig.	Sig.	Sig.	Sig.
AAA <0.001	AAA 0.435	TAVI <0.001	TAVI 0.435	TAVI <0.001	TAVI <0.001
TA <0.001	TA <0.001	TA <0.001	TA <0.001	AAA <0.001	AAA <0.001
**Lumbar spine L4 level measurements for average psoas muscle density in Houndsfield units**					
36.4 (10.7)	39.5 (11.6)	35.6 (12.8)	34.7 (14.6)	44.3 (31.7)	39.3 (11.5)
Sig.	Sig.	Sig.	Sig.	Sig.	Sig.
AAA 0.435	AAA <0.001	TAVI 0.435	TAVI <0.001	TAVI <0.001	TAVI 0.984
TA <0.001	TA 0.984	TA <0.001	TA 0.010	AAA <0.001	AAA 0.010

### The associations of different indices of sarcopenia and mortality

The analysis of all three cohorts showed that almost all psoas measurements were associated with mortality risk among men, but not among women. (Table 3. and Table 4). Overall, the effect sizes for the associations between sarcopenia indices and mortality were very similar, regardless of the anatomical lumbar level from which they were measured (Table 3).

**Table 3.** Linear associations between psoas muscle parameters and mortality in a meta-analysis of three independent patient populations in men.

	Hazard Ratio for Long-term Mortality				
	Fixed effects	Sig.	Random effects	Sig.	I^2^
L3 Level measurement					
PMA	0.856 (0.781–0.938)	0.001	0.850 (0.767–0.943)	0.002	0.27
PMD	0.845 (0.780–0.915)	<0.001	0.827 (0.689–0.992)	0.041	0.66
PMA/height	0.862 (0.788–0.944)	0.001	0.853 (0.764–0.952)	0.005	0.23
PMA/BSA	0.873 (0.801–0.952)	0.002	0.848 (0.740–0.971)	0.017	0.49
sLPMA	0.792 (0.726–0.865)	<0.001	0.767 (0.644–0.914)	0.003	0.60
pLPMA	0.792 (0.723–0.868)	<0.001	0.792 (0.723–0.868)	<0.001	0.57
L4 level measurement					
PMA	0.846 (0.772–0.927)	0.002	0.864 (0.772–0.927)	0.002	0
PMD	0.846 (0.781–0.917)	<0.001	0.828 (0.651–1.053)	0.124	0.80
PMA/height	0.851 (0.777–0.932)	0.001	0.851 (0.777–0.932)	0.001	0
PMA/BSA	0.863 (0.792–0.941)	0.006	0.854 (0.767–0.950)	0.004	0.20
sLPMA	0.795 (0.729–0.868)	<0.001	0.778 (0.644–0.941)	0.009	0.67
pLPMA	0.793 (0.725–0.869)	<0.001	0.774 (0.653–0.942)	0.01	0.65

Abbreviations: PMA: Psoas muscle area; PMD Psoas muscle density; BSA: body-surface area; sLPMA, Sum score of Lean Psoas Muscle Mass; pLPMA: product score of lean Psoas

**Table 4.** Linear associations between psoas muscle parameters and mortality in a meta-analysis of three independent patient populations in women.

	Hazard Ratio for Long-term Mortality				
	Fixed effects	Sig.	Random effects	Sig.	I^2^
L3 Level measurement					
PMA	0.934(0.820–1.065)	0.307	0.940(0.804–1.099)	0.438	0.17
PMD	0.949(0.843–1.068)	0.382	0.949(0.843–1.068)	0.382	0
PMA/height	0.936(0.820–1.067)	0.321	0.936(0.820–1.067)	0.321	0
PMA/BSA	1.001(0.881–1.137)	0.987	1.004(0.875–1.153)	0.949	0
sLPMA	0.914(0.806–1.037)	0.164	0.914(0.806–1.037)	0.164	0
pLPMA	0.944(0.829–1.075)	0.384	0.944(0.829–1.075)	0.384	0
L4 level measurement					
PMA	0.888(0.778–1.012)	0.075	0.918(0.775–1.116)	0.391	0.28
PMD	0.935(0.835–1.046)	0.242	0.934(0.831–1.048)	0.3245	0
PMA/height	0.887(0.778–1.012)	0.075	0.908(0.762–1.081)	0.278	0.09
PMA/BSA	0.957(0.841–1.090)	0.512	0.999(0.818–1.219)	0.989	0.35
sLPMA	0.883(0.781–0.998)	0.047	0.883(0.781–0.998)	0.047	0
pLPMA	0.904(0.789–1.025)	0.115	0.904(0.789–1.025)	0.115	0

Abbreviations: PMA: Psoas muscle area; PMD: Psoas muscle density; BSA: body-surface area; sLPMA: sum score of lean psoas muscle mass; pLPMA: product score of lean Psoas.

In terms of hazard ratios (for death) in men, the magnitude of the association ranged between 0.873 (0.801–0.952, *p* = 0.002 for fixed effects model) for PMA indexed to BSA at the L3 level and 0.767 (0.644–0.914, *p* = 0.003 for fixed effects model) for lean PMA defined by the sum score of PMA and PMD at level 3(Table 3). There was very little difference in the association between PMA and PMD and mortality (HR 0.83, 95% CI 0.69–0.99, *p* = 0.002 and HR 0.85, 95% CI 0.77–0.94, *p* = 0.041 for one SD increase in PMA and PMD in the random effects model), and indexing to body height or BSA did not change the results significantly. High heterogeneity was observed between the cohorts (I^2^ values > 0.5, 6/12 analyses)(Table 3).

Observing the cohort-specific HRs, the greatest magnitude for the association was observed among men undergoing surgery for thoracic aorta and aortic valve pathology (HR estimates ranging between 0.502 and 0.732, with the strongest association for lean PMA defined as the product score of PMA and PMD from the L3 level: HR 0.502 with 95% CI from 0.316–0.796, *p* = 0.003) (Supplementary Table 2). The weakest magnitude of the association was observed in men of the AAA cohort with HR estimates ranging between 0.758 and 0.919, and the strongest association with mortality observed for lean PMA was defined as the sum score of PMA and PMD from L4 level (HR 0.758 with 95% CI 0.677–0.848) (Supplementary Table 2).

Among women, where no significant linear associations between psoas muscle parameters and mortality were observed, there was very little heterogeneity in the results, as all cohorts showed nonsignificant results and only the sum term of PMA and PMD seemed to show a statistically significant association with mortality (Table 4 and Supplementary Table 3). The predictive values of psoas muscle parameters and age in predicting mortality were modest among men and very weak among women. The AUC values ranged between 0.679–0.660 among men (Table 5) and 0.604–0.583 among women (Table 6).

**Table 5.** Predictive value of psoas muscle parameters combined with age for long-term mortality in a meta-analysis of three different patient population in men.

	AUC value for Long-term Mortality		
	Fixed effects	Random effects	I^2^
L3 Level measurement			
PMA	0.654(0.625–0.683)	0.666(0.584–0.748)	0.82
PMD	0.661(0.632–0.690)	0.674(0.576–0.772)	0.86
PMA/height	0.652(0.623–0.681)	0.664(0.585–0.743)	0.81
PMA/BSA	0.652(0.623-–0.681)	0.665(0.579–0.751)	0.83
sLPMA	0.663(0.634–0.692)	0.677(0.576–0.778)	0.87
pLPMA	0.662(0.633–0.691)	0.675(0.577–0.773)	0.87
L4 level measurement			
PMA	0.652(0.623–0.681)	0.663(0.584–0.742)	0.81
PMD	0.667(0.638–0.695)	0.678(0.582–0.775)	0.86
PMA/height	0.650(0.621–0.679)	0.661(0.584–0.738)	0.80
PMA/BSA	0.648(0.619–0.677)	0.660(0.578–0.743)	0.82
sLPMA	0.668(0.639–0.696)	0.679(0.578–0.780)	0.88
pLPMA	0.667(0.638–0.696)	0.679(0.578–0.780)	0.88

Abbreviations: PMA: Psoas muscle area; PMD: Psoas muscle density; BSA: body-surface area; sLPMA: sum score of lean psoas muscle mass; pLPMA: product score of lean psoas.

**Table 6.** Predictive value of psoas muscle parameters combined with age for long-term mortality in a meta-analysis of three different patient population in women.

	AUC value for Long-term Mortality		
	Fixed effects	Random effects	I^2^
L3 Level measurement			
PMA	0.595(0.551–0.640)	0.595(0.551–0.640)	0
PMD	0.601(0.556–0.646)	0.609(0.546–0.672)	0.25
PMA/height	0.595(0.551–0.639)	0.595(0.551–0.639)	0
PMA/BSA	0.587(0.542–0.631)	0.587(0.542–0.631)	0
sLPMA	0.590(0.545–0.634)	0.590(0.545–0.634)	0
pLPMA	0.583(0.538–0.628)	0.583(0.538–0.628)	0
L4 level measurement			
PMA	0.598(0.554–0.642)	0.598(0.554–0.642)	0
PMD	0.604(0.560–0.649)	0.615(0.550–0.679)	0
PMA/height	0.599(0.555-0.643)	0.599(0.555–0.643)	0
PMA/BSA	0.589(0.544–0.634)	0.589(0.544–0.634)	0
sLPMA	0.593(0.548–0.637)	0.593(0.548–0.637)	0
pLPMA	0.591(0.546–0.636)	0.591(0.546–0.636)	0

Abbreviations: PMA: Psoas muscle area; PMD: Psoas muscle density; BSA: body-surface area; sLPMA: sum score of lean psoas muscle mass; pLPMA: Product score of Lean Psoas.

## Discussion

This retrospective analysis of three independent cohorts of patients undergoing vascular and valvular interventions is the first to provide accurate data on the associations between different psoas muscle parameters depicting possible sarcopenia and long-term mortality.

Based on our observations, psoas muscle parameters were significantly associated with mortality among men, but not among women. The sum score or the product score of PMA and PMD showed the strongest magnitude for the linear association, which is not surprising, as these combined variables incorporate information from two different parameters into one variable. In men, the heterogeneity across the cohorts was very high for most associations. The predictive value of all muscle parameters was modest at best and strikingly similar regardless of the muscle parameters used in the analysis, and of the anatomical level used for measurement.

Given the large sample size of the present study, we also had the opportunity to evaluate the results separately among men and women. This topic is important in this context for a couple of reasons. First, women are usually underrepresented in patients with vascular or cardiovascular disease. Second, the analyses may be confounded by different factors such as selection bias of women undergoing invasive operations (women may be subjected to more stringent selection prior to invasive operations which may cause survival bias compared to men) [35] and sex-related differences in anthropometry with women having lower values of muscle mass indexed to body size and different body composition [36, 37]. Additionally, hormonal differences may affect the rate of muscle loss after significant interventions and the progression of sarcopenia, potentially leading to different prognostic implications. According to our results, the predictive value of the muscle parameters was poor among women and the linear associations were not significant nor strong when evaluated by the regression coefficients and the resulting hazard ratios. Based on our data we are unable to explain this difference between men and women in the association between psoas muscle status and mortality. Despite some earlier conflicting findings [17], a retrospective analysis of a large consecutive series of over nine hundred women undergoing TAVI found no association between PMA (indexed to height) and long-term mortality supporting our finding that the linear association, is most likely very weak or non-existent [38].

Numerous previous scientific publications have shown that psoas muscle measurements are associated with overall mortality after various invasive interventions. The foremost problem of many studies is that they have relied on results obtained from very specific settings, using arbitrary study-specific cutoff values for muscle parameters and numerous differently indexed muscle parameters. In several populations undergoing cardiologic, cardio-thoracic, or vascular surgical interventions or surgery of the abdominal area, simple cross-sectional area of a psoas muscle or psoas muscle area indexed to height have been shown to associate with mortality [4, 5, 11–13, 22, 23, 27]. In some of these studies, measurements were made at the L3 level [12, 23, 27] and in others at the L4 level [4, 5, 11, 13, 22]. According to our results, the level of measurement is not important and indexing to body height or size does not seem to confer any additional benefit. Positive publication bias, small sample sizes, and multiple testing issues are probable explanations for heterogeneity in previously published results [4, 5, 7, 11–13, 15, 17–23, 26–32].

In some recent studies, psoas muscle density has been shown to predict mortality, and it is believed to provide a better depiction of the status of musculature because intramuscular fat is also accounted for [7, 28–30]. Combining absolute values PMA and PMD to one metric has been shown to associate with mortality among AAA patients [15, 32]. However, these results are mostly anecdotal, because the populations from which the observations were drawn are too small for any qualitative comparisons between different muscle parameters that are very closely intercorrelated. Furthermore, producing an arbitrary product term from two variables with very different numerical scales (i.e. non-standardized values) can lead to spurious results. Nevertheless, it appears that the product of these two correlating variables performs well since both measurements of the equation are associated with mortality and provide different types of information on the quantity and quality of psoas muscles. In the present study, we also formed a combination of these two variables after scaling the variables. According to our results, the magnitude of the association seems somewhat stronger in terms of the observed hazard ratio value for the linear association between the product term or sum term of PMA and PMD, and mortality. The problem with forming this type of estimate of ‘lean’ muscle mass is that there is no actual physical scale for it and such it is impossible to determine an absolute cut-off for this metric that would indicate significant sarcopenia. More research is required to determine if the value of these metrics exceed these methodological challenges.

According to our results, the linear associations between psoas muscle measurements and mortality were observed significant among men but according to area under curve analysis, they were not generally strong predictors of mortality. However, it is possible that the association between these muscle parameters and mortality risk is nonlinear, but currently, there is no well-defined threshold for significant sarcopenia. For any further conclusion regarding the possible deviation from nonlinearity, analysis in larger patient sets with appropriate independent validation is required. Unfortunately, we also lack information of the actual functional status of our patients. The possible interplay between objectively measured muscle strength and functional capacity, which are the foremost measures to assess possible sarcopenia, and radiographically measurable muscle status is clearly a subject for further studies [9].

Our results are based on age adjusted analysis and although we did not find any significant change in the results after adjusting additionally for renal function (or indexing PMA to body height and BSA), our results do not indicate that psoas muscle status could be used to replace existing preoperative evaluation methods when assessing surgical risk. The purpose of our work was to observe the differences in the association between long-term mortality and different indices of psoas muscle status. Due to the heterogenous patient populations used in our meta-analysis, we did not have uniformly recorded data of risk scores such as the EuroScore or STS score. For this reason, we could not adjust the analyses similarly for surgical risk in each cohort. The possible independent predictive power of any psoas muscle indices should be evaluated separately in each patient population before their true value can be considered in clinical risk evaluation.

To the best of our knowledge, this type of comprehensive study of the possible superiority or lack thereof of psoas muscle measurements in mortality prediction has not been carried out before. This study included over two thousand patients and thus had a high statistical power compared to previously published studies on the subject. We were able to combine data from three distinct patient groups undergoing cardiovascular intervention. This increased the generalizability of the results. Although this method introduces heterogeneity to the results, it provides a more objective perspective on the performance of these measurement methods for muscle status (or sarcopenia). Finally, although the present meta-analysis is based on retrospective registries instead of prospective trials dedicated to this hypothesis, the main exposure variables can be measured with high accuracy also retrospectively.

## Conclusion

This study showed that no single muscle parameter was superior to others in predicting long-term mortality. The linear association between muscle parameters and mortality was clear among men, but not among women. Combining the psoas muscle area with density to form a lean estimate of the psoas muscle area appears to be strongly associated with mortality.

## Supplementary Material

Supplemental Material

## Data Availability

The data that support the findings of this study are available in anonymized form from the corresponding author [JAH] upon reasonable request.
