# Supplementary material for: Psoas muscle quantified muscle status and long-term mortality after cardiovascular interventions
Source: Ann Med. 2023 Sep 22;55(2):2259798. doi: 10.1080/07853890.2023.2259798 (PMC10519254; doi:10.1080/07853890.2023.2259798)
Supplement: Supplemental Material [file IANN_A_2259798_SM1745.docx]

|  | AAA (n=828) | TAVI (n=983) | TAA (n=437) |
| --- | --- | --- | --- |
| Age | 0 | 0 | 0 |
| Gender | 0 | 0 | 0 |
| Height | 0 | 0 | 0 |
| BSA | 0 | 0 | 0 |
| L3 area | 1 | 1 | 0 |
| L3 density | 0 | 0 | 0 |
| L4 area | 256 | 0 | 10 |
| L4 density | 254 | 1 | 10 |

Supplementary Table 1. Cohort specific numbers of patients with missing values that had to be imputed.

Supplementary Table 2. Cohort specific hazard ratio estimates for the association between psoas muscle parameters and mortality

|  | MEN | | | WOMEN | | | FIXED EFFECTS | RANDOM EFFECTS | I^2^ |
| --- | --- | --- | --- | --- | --- | --- | --- | --- | --- |
|  | AAA | TAVI | TAA | AAA | TAVI | TAA | ALL | ALL | ALL |
| L3 Level measurement |  |  |  |  |  |  |  |  |  |
| PMA | 0.895(0.799-1.002) | 0.816(0.619-0.963) | 0.645(0.428-0.972) | 1.119(0.848-1.476) | 0.900(0.769-1.052) | 0.779(0.485-1.251) | 0.881(0.817-0.949) | 0.881(0.817-0.944) | 0.208476561 |
| PMD | 0.821(0.743-0.907) | 0.953(0.823-1.104) | 0.631(0.453-0.879) | 0.888(0.700-1.126) | 0.963(0.835-1.111) | 1.037(0.646-1.666) | 0.876(0.820-0.936) | 0.881(0.801-0.969) | 0.436843253 |
| PMA/height | 0.903(0.807-1.009) | 0.813(0.687-0.961) | 0.677(0.457-1.002) | 1.098(0.830-1.452) | 0.906(0.775-1.060) | 0.789(0.491-1.268) | 0.885(0.822-0.954) | 0.885(0.822-0.954) | 0.092698535 |
| PMA/BSA | 0.919(0.826-1.023) | 0.820(0.700-0.960) | 0.641(0.435-0.947) | 1.186(0.896-1.570) | 0.963(0.828-1.120) | 0.910(0.579-1.430) | 0.911(0.848-0.979) | 0.910(0.829-0.999) | 0.430542908 |
| sLPMA | 0.779(0.695-0.873) | 0.861(0.743-0.997) | 0.554(0.372-0.796) | 0.965(0.730-1.275) | 0.905(0.780-1.050) | 0.865(0.536-1.395) | 0.830(0.772-0.892) | 0.832(0.759-0.912) | 0.418519907 |
| pLPMA | 0.782(0.695-0.880) | 0.851(0.731-0.992) | 0.502(0.316-0.796) | 0.993(0.747-1.319) | 0.930(0.798-1.083) | 0.952(0.591-1.532) | 0.840(0.779-0.905) | 0.844(0.764-0.933) | 0.474409725 |
|  |  |  |  |  |  |  |  |  |  |
| L4 level measurement |  |  |  |  |  |  |  |  |  |
| PMA | 0.872(0.778-0.978) | 0.819(0.694-0.965) | 0.701(0.467-1.053) | 1.096(0.819-1.468) | 0.830(0.711-0.970) | 0.939(0.600-1.471) | 0.859(0.979-0.926) | 0.859(0.797-0.926) | 0 |
| PMD | 0.800(0.725-0.883) | 1.017(0.876-1.181) | 0.632(0.449-0.890) | 0.855(0.688-1.061) | 0.981(0.854-1.126) | 0.831(0.532-1.299) | 0.875(0.820-0.934) | 0.873(0.773-0.986) | 0.622673038 |
| PMA/height | 0.881(0.788-0.985) | 0.810(0.684-0.960) | 0.732(0.498-1.075) | 1.061(0.795-1.417) | 0.833(0.712-0.975) | 0.957(0.613-1.494) | 0.863(0.800-0.930) | 0.863(0.800-0.930) | 0 |
| PMA/BSA | 0.903(0.812-1.004) | 0.812(0.692-0.951) | 0.695(0.475-1.018) | 1.169(0.873-1.564) | 0.888(0.760-1.037) | 1.094(0.722-1.657) | 0.891(0.829-0.957) | 0.891(0.829-0.957) | 0.3114203943 |
| sLPMA | 0.758(0.677-0.848) | 0.904(0.780-1.047) | 0.587(0.401-0.859) | 0.900(0.688-1.177) | 0.879(0.760-1.018) | 0.870(0.567-1.336) | 0.824(0.767-0.884) | 0.827(0.751-0.911) | 0.3659096047 |
| pLPMA | 0.760(0.677-0.853) | 0.894(0.767-1.042) | 0.538(0.342-0.845) | 0.921(0.706-1.200) | 0.899(0.774-1.044) | 0.905(0.567-1.444) | 0.830(0.771-0.893) | 0.835(0.755-0.923) | 0.4113369762 |

Supplementary Table 3. Cohort specific C-statistic values for the predictive value of age and muscle parameters for mortality

|  | MEN | | | WOMEN | | | FIXED EFFECTS | RANDOM EFFECTS | I^2^ |
| --- | --- | --- | --- | --- | --- | --- | --- | --- | --- |
|  | AAA | TAVI | TAA | AAA | TAVI | TAA | ALL | ALL | ALL |
| L3 Level measurement |  |  |  |  |  |  |  |  |  |
| PMA | 0.652(0.613-0.691) | 0.604(0.551-0.656) | 0.753(0.681-0.824) | 0.586(0.469-0.704) | 0.597(0.546-0.648) | 0.597(0.459-0.736) | 0.636(0.612-0.660) | 0.636(0.585-0.687) | 0.67917001 |
| PMD | 0.672(0.633-0.711) | 0.590(0.538-0.643) | 0.770(0.692-0.849) | 0.689(0.574-0.804) | 0.585(0.534-0.637) | 0.585(0.441-0.729) | 0.643(0.619-0.667) | 0.649(0.588-0.709) | 0.775611675 |
| PMA/height | 0.650(0.611-0.690) | 0.604(0.551-0.656) | 0.749(0.677-0.820) | 0.588(0.471-0.704) | 0.597(0.546-0.648) | 0.592(0.458-0.726) | 0.635(0.611-0.659) | 0.635(0.585-0.684) | 0.662894688 |
| PMA/BSA | 0.649(0.609-0.688) | 0.601(0.548-0.653) | 0.756(0.685-0.827) | 0.582(0.464-0.6999 | 0.590(0.539-0.642) | 0.571(0.435-0.706) | 0.633(0.609-0.657) | 0.631(0.576-0.686) | 0.717491634 |
| sLPMA | 0.675(0.637-0.714) | 0.590(0.538-0.643) | 0.775(0.697-0.853) | 0.592(0.470-0.714) | 0.589(0.538-0.640) | 0.590(0.450-0.730) | 0.642(0.617-0.666) | 0.639(0.577-0.701) | 0.782486987 |
| pLPMA | 0.675(0.637-0.714) | 0.590(0.537-0.642) | 0.770(0.690-0.850) | 0.557(0.434-0.681) | 0.588(0.537-0.640) | 0.573(0.432-0.713) | 0.639(0.614-0.663) | 0.632(0.567-0.696) | 0.787192999 |
|  |  |  |  |  |  |  |  |  |  |
| L4 level measurement |  |  |  |  |  |  |  |  |  |
| PMA | 0.655(0.615-0.694) | 0.599(0.547-0.651) | 0.746(0.673-0.819) | 0.587(0.469-0.705) | 0.602(0.552-0.653) | 0.579(0.442-0.716) | 0.636(0.612-0.660) | 0.634(0.585-0.683) | 0.655457258 |
| PMD | 0.678(0.639-0.716) | 0.595(0.542-0.648) | 0.772(0.695-0.848) | 0.692(0.579-0.806) | 0.587(0.536-0.638) | 0.601(0.462-0.740) | 0.648(0.624-0.672) | 0.653(0.593-0.713) | 0.779508950 |
| PMA/height | 0.652(0.613-0.692) | 0.599(0.547-0.652) | 0.742(0.670-0.815) | 0.592(0.475-0.709) | 0.604(0.553-0.654) | 0.578(0.441-0.715) | 0.635(0.611-0.659) | 0.634(0.586-0.681) | 0.631418651 |
| PMA/BSA | 0.649(0.610-0.689) | 0.595(0.543-0.648) | 0.747(0.674-0.820) | 0.553(0.432-0.675) | 0.595(0.544-0.646) | 0.594(0.447-0.740) | 0.631(0.607-0.655) | 0.629(0.575-0.682) | 0.687503335 |
| sLPMA | 0.682(0.643-0.720) | 0.589(0.537-0.642) | 0.774(0.699-0.849) | 0.603(0.480-0.726) | 0.591(0.540-0.643) | 0.590(0.455-0.725) | 0.646(0.622-0.670) | 0.642(0.580-0.704) | 0.796061277 |
| pLPMA | 0.682(0.643-0.720) | 0.589(0.537-0.641) | 0.773(0.697-0.849) | 0.601(0.478-0.742) | 0.590(0.539-0.642) | 0.582(0.443-0.720) | 0.645(0.621-0.669) | 0.641(0.578-0.703) | 0.796263078 |
